# Dr. Henry Nichell

**Published:** 1901-03

**Authors:** 


### OBITUARY NOTES.

Dr. Henry Nichell, U. of B. 1857, died at his residence, 27 Day's Park, Buffalo, Thursday, February 14, 1901, in the eightieth year of his age. Dr. Nichell was well known especially to the older residents of this city as a conscientious physician and kind-hearted man.

